# Obesity and eating habits among college students in Saudi Arabia: a cross sectional study

**DOI:** 10.1186/1475-2891-9-39

**Published:** 2010-09-19

**Authors:** Abdallah S Al-Rethaiaa, Alaa-Eldin A Fahmy, Naseem M Al-Shwaiyat

**Affiliations:** 1 Department of Clinical Laboratory, College of Health Sciences at Rass, Qassim University, Saudi Arabia; 2 Department of Anatomy, Faculty of Medicine, Ain Shams University, Egypt; 3 Department of Clinical Nutrition, College of Health Sciences at Rass, Qassim University, Saudi Arabia

## Abstract

**Background:**

During the last few decades, the Kingdom of Saudi Arabia (KSA) experienced rapid socio-cultural changes caused by the accelerating economy in the Arabian Gulf region. That was associated with major changes in the food choices and eating habits which, progressively, became more and more "Westernized". Such "a nutritional transition" has been claimed for the rising rates of overweight and obesity which were recently observed among Saudi population. Therefore, the objectives of the current work were to 1) determine the prevalence of overweight and obesity in a sample of male college students in KSA and 2) determine the relationship between the students' body weight status and composition and their eating habits.

**Methods:**

A total of 357 male students aged 18-24 years were randomly chosen from College of Health Sciences at Rass, Qassim University, KSA for the present study. A Self-reported questionnaire about the students' eating habits was conducted, and their body mass index (BMI), body fat percent (BF%), and visceral fat level (VFL) were measured. Data were analyzed using SPSS statistical software, and the Chi-square test was conducted for variables.

**Results:**

The current data indicated that 21.8% of the students were overweight and 15.7% were obese. The total body fat exceeded its normal limits in 55.2% of the participants and VFL was high in 21.8% of them. The most common eating habits encountered were eating with family, having two meals per day including breakfast, together with frequent snacks and fried food consumption. Vegetables and fruits, except dates, were not frequently consumed by most students. Statistically, significant direct correlations were found among BMI, BF% and VFL (P < 0.001). Both BMI and VFL had significant inverse correlation with the frequency of eating with family (P = 0.005 and 0.007 respectively). Similar correlations were also found between BMI and snacks consumption rate (P = 0.018), as well as, between VFL and the frequency of eating dates (P = 0.013).

**Conclusions:**

Our findings suggest the need for strategies and coordinated efforts at all levels to reduce the tendency of overweight, obesity and elevated body fat, and to promote healthy eating habits in our youth.

## Background

Obesity is often defined as a condition of abnormal and excessive fat accumulation in adipose tissue to the extent that health may be adversely affected [1]. The prevalence of obesity is increasing worldwide at an alarming rate in both developing and developed countries. It has become a serious epidemic health problem, estimated to be the fifth leading cause of mortality at global level [2]. Moreover, it is a risk factor for many diseases such as certain cancers, hypertension, type II diabetes mellitus, dyslipidemia, metabolic syndrome and coronary heart disease [3-6]. The rapid cultural and social changes that have occurred in the Arabian Gulf region, since the discovery of oil and the economic boom during the 1970's and 1980's, were associated with an alarming increase in obesity [7-11]. One of the major causes of obesity is the changes in the diet, in terms of quantity and quality, which has become more "Westernized" [12]. In the Kingdom of Saudi Arabia (KSA), recent studies revealed increasing consumption of animal products and refined foods in the diet at the expense of vegetables and fruits [13,14]. These dietary changes were accused for increasing the prevalence of both overweight and obesity observed among Saudi children, adolescences and adults in the last few decades [15-18].

College students are highly exposed to unhealthy eating habits leading to body weight gain [19]. According to WHO, obesity is generally more common among women than men [1]. However, studies on college students revealed higher rates of obesity in males than in females [19,20]. In KSA, Rasheed et al [21] documented that 30.6% of female health college students were either overweight or obese. In contrast, no study was found in our literature search regarding obesity prevalence in Saudi male college students. Therefore, the aim of the current work is to assess overweight and obesity rates among male college students in KSA and to correlate their body weight status and composition with their eating habits.

## Methods

### Design and Sample

A cross sectional study was conducted in College of Health Sciences at Rass, Qassim University, KSA during the spring semester of 2009. A total number of 357 male students aged 18-24 years had participated in the study after signing a written consent form according to Helsinki Declaration. All of the participants were Saudi of the Arabian ethnicity and were chosen by the stratified random sampling method by department and class year. The response rate among students was 96.5%. The study was approved by the committee of research ethics in Qassim University.

### Data Collection

Self-reported questionnaire and anthropometric measurements were used for data collection. The questionnaire was designed to study eating, drinking, and smoking habits among college students, and its use in that respect had been standardized [20,22,23]. Prior to filling out the questionnaire, the students were informed about the study and were given instructions on how to fill out the questionnaire completely and truthfully. Anthropometric measurements including height, weight, body mass index (BMI), body fat percent (BF%), and visceral fat level (VFL), were conducted. Weight, BMI, BF%, and VFL were determined using a bioelectrical impedance analysis (BIA) device: Omron body composition monitor (BF500, Omron Healthcare Co. Ltd., Kyoto, Japan) [24]. As fluctuations in body water content affect body composition, measurements were taken in the morning when the students remain without eating, drinking, bathing or exercising for at least two hours. Following the manufacturer's instructions, students were asked to wipe off the sole of their feet before stepping onto the measuring platform as unclean foot pads interfere with the device conductivity. Digital Physician Scale (MDW-250L, Adam Equipment Co. Ltd., UK) was used for measuring height, where students were asked to take off their shoes and socks and stand straight, with the head in the Frankfurt plane, feet together, knees straight, and heels, buttocks, and shoulder blades are in contact with the vertical surface of the scale [25].

Body mass index (BMI), which is the ratio of weight in kilogram to height in meter square, was used to assess body weight status. According to the National Institutes of Health (NIH), adults were classified based on their BMI to underweight (BMI < 18.5), normal (BMI = 18.5-24.9), overweight (BMI = 25-29.9), or obese (BMI ≥ 30). Furthermore, obesity was subdivided to three grades: Grade 1 (BMI = 30-34.9), Grade 2 (BMI = 35-39.9) and Grade 3 or extreme obesity (BMI ≥ 40) [26]. Taking in consideration age and gender, the participants were classified as having low (BF% < 8), normal (BF% = 8-19.9), high (BF% = 20-24.9), or very high (BF% ≥ 25) body fat [27]. Visceral fat was also measured and the students were categorized as having normal (VFL = 1-9) or high (VFL = 10-30) visceral fat [28].

### Data Analysis

The Statistical Package for Social Sciences (SPSS Inc., Chicago, IL, USA) version 17 was used for data analysis. Results were expressed as means ± standard deviations. All of the analyzed variables were non-parametric and were tested using Chi-squared tests. All reported *P *values were made on the basis of two-tailed tests. Differences were considered statistically significant at P value < 0.05.

## Results

### Students' characteristics

A total of 357 male students, with an average age of 20.4 ± 1.3 years, participated in the current study. The mean weight and height of the students were respectively 69.9 ± 15.6 kg and 168.8 ± 6.1 cm. The average BMI, BF% and VFL of the participants were respectively 24.6 ± 5.2, 22.4 ± 9.2 and 6.5 ± 4.3 (Table 1).

**Table 1:** Characteristics of the participants (means ± SD).

Variable	Total
Number of Students	357

Age (years)	20.4 ± 1.3

Weight (kg)	69.9 ± 15.6

Height (cm)	168.8 ± 6.1

BMI (kg/m^2^)	24.6 ± 5.2

BF%	22.4 ± 9.2

VFL (1-30)	6.5 ± 4.3

### Anthropometry

The measurements of BMI indicated that the majority of students (57.4%) had normal weight. Overweight and obese subjects represented 21.8% and 15.7% of the students respectively, compared to 5.0% for underweight subjects. Most of the obese students (11.5% of all participants) fall in grade 1 obesity whereas, grade 2 and grade 3 obese students represented 3.4% and 0.8% of the whole sample, respectively (Table 2). Based on BF%, 41.7% of the students had normal body fat, which was low in 3.1%, high in 16.8% and very high in 38.4% of them (Table 3). Visceral fat was normal in more than three fourth of the participants (78.2%) (Table 4). The correlations among these anthropometric measurements (BMI, BF% and VFL) were found to be direct and significant (P < 0.001). Body fat exceeded its normal limits not only in all overweight and obese subjects, but also in 30.7% of those with normal BMI. In contrast, high levels of visceral fat were observed in all obese and in 28.2% of the overweight students. Moreover, these high VFL's were confined to 56.9% of students with very high BF% (Table 5).

**Table 2:** Prevalence of obesity among participants based on BMI categories [Total (Percent)] and their mean and range BF% and VFL.

BMI categories	Total (Percent)	BMI	BF%		VFL	
		
		mean ± SD	mean ± SD	range	mean ± SD	range
**Underweight**	N = 18 (5.0%)	17.9 ± 0.4	8.2 ± 1.2	6.5-10.1	1.1 ± 0.2	1-2

**Normal**	N = 205 (57.4%)	21.6 ± 1.9	17.3 ± 4.8	5.5-28.5	4.0 ± 1.8	1-7

**Overweight**	N = 78 (21.8%)	27.2 ± 1.4	28.6 ± 2.9	22.5-36.0	9.0 ± 1.1	7-12

**Obese**	N = 56 (15.7%)	34.0 ± 3.5	37.1 ± 4.0	29.8-46.5	14.1 ± 2.3	11-20

**Obese grade 1**	N = 41 (11.5%)	32.1 ± 1.4	35.4 ± 2.7	29.8-41.7	12.9 ± 1.1	11-15

**Obese grade 2**	N = 12 (3.4%)	37.9 ± 1.4	40.6 ± 3.0	35.5-44.6	16.6 ± 1.0	15-18

**Obese grade 3**	N = 3 (0.8%)	43.3 ± 1.0	45.5 ± 1.0	44.6-46.5	20 ± 0.0	20-20

**Table 3:** Prevalence of obesity based on BMI categories cross tabulated with BF% categories [Total (Percent)].

BMI categories	BF% categories*				Total (Percent)
		
	Low	Normal	High	Very High	
**Underweight**	N = 8 (2.2%)	N = 10 (2.8%)	N = 0 (0.0%)	N = 0 (0.0%)	N = 18 (5.0%)

**Normal**	N = 3 (0.8%)	N = 139 (38.9%)	N = 52 (14.6%)	N = 11 (3.1%)	N = 205 (57.4%)

**Overweight**	N = 0 (0.0%)	N = 0 (0.0%)	N = 8 (2.2%)	N = 70 (19.6%)	N = 78 (21.8%)

**Obese**	N = 0 (0.0%)	N = 0 (0.0%)	N = 0 (0.0%)	N = 56 (15.7%)	N = 56 (15.7%)

**Total (Percent)**	N = 11 (3.1%)	N = 149 (41.7%)	N = 60 (16.8%)	N = 137 (38.4%)	N = 357 (100%)

* P < 0.001

**Table 4:** Prevalence of obesity based on BMI categories cross tabulated with VFL categories [Total (Percent)].

BMI categories	VFL categories*		Total (Percent)
		
	Normal	High	
**Underweight**	N = 18 (5.0%)	N = 0 (0.0%)	N = 18 (5.0%)

**Normal**	N = 205 (57.4%)	N = 0 (0.0%)	N = 205 (57.4%)

**Overweight**	N = 56 (15.7%)	N = 22 (6.2%)	N = 78 (21.8%)

**Obese**	N = 0 (0.0%)	N = 56 (15.7%)	N = 56 (15.7%)

**Total (Percent)**	N = 279 (78.2%)	N = 78 (21.8%)	N = 357 (100%)

* P < 0.001

**Table 5:** BF% categories cross tabulated with VFL categories [Total (Percent)].

BF% categories	VFL categories*		Total (Percent)
		
	Normal	High	
**Low**	N = 11 (3.1%)	N = 0 (0.0%)	N = 11 (3.1%)

**Normal**	N = 149 (41.7%)	N = 0 (0.0%)	N = 149 (41.7%)

**High**	N = 60 (16.8%)	N = 0 (0.0%)	N = 60 (16.8%)

**Very High**	N = 59 (16.5%)	N = 78 (21.8%)	N = 137 (38.4%)

**Total (Percent)**	N = 279 (78.2%)	N = 78 (21.8%)	N = 357 (100%)

* P < 0.001

### Eating habits

Although irregular meals consumption was reported in 63.3% of students, the vast majority of them (88.6%) have breakfast at least three times per week. Most of the participants (55.7%) eat two meals per day, while 31.4% of them eat three meals. Eating snacks was a common habit among students and its daily consumption was reported in 31.7% of them. With the exception of dates which are taken at least three times weekly by 60.5% of students, vegetables and fruits were not frequently consumed. In fact the percentage of students who rarely eat vegetables and fruits were respectively 32.2 and 36.1, and those who eat them once or twice per week were 32.2% and 40.3%. Almost half of participants (46.8%) eat fried foods at least thrice a week. Sharing meals with family was a common habit among the students; 66.4% of them eat daily with their families. In addition, the majority of students (59.7%) was aware of the types of food they should eat in order to have a balanced nutrition. We also found that 86.8% of the students were non-smokers and 95.8% of them never drink alcohol (Table 6).

**Table 6:** Participants response for eating, drinking and smoking habits questionnaire.

Questions Asked	Answer Levels		Total (Percent)
Q1. Do you take your meals regularly?	A.	Always regular	N = 131 (36.7%)
	B.	Irregular	N = 226 (63.3%)

Q2. Do you take breakfast?	A.	Daily	N = 178 (49.9%)
	B.	Three or four times per week	N = 138 (38.7%)
	C.	Once or twice per week	N = 29 (8.1%)
	D.	Rarely	N = 12 (3.4%)

Q3. How many times do you eat meals except snacks?	A.	One time	N = 40 (11.2%)
	B.	Two times	N = 199 (55.7%)
	C.	Three times	N = 112 (31.4%)
	D.	Four times	N = 6 (1.7%)

Q4. How often do you take snacks apart from regular meals?	A.	Daily	N = 113 (31.7%)
	B.	Three or four times per week	N = 89 (24.9%)
	C.	Once or twice per week	N = 85 (23.8%)
	D.	Rarely	N = 70 (19.6%)

Q5. How often do you eat green, red or yellow colored vegetables?	A.	Daily	N = 40 (11.2%)
	B.	Three or four times per week	N = 87 (24.4%)
	C.	Once or twice per week	N = 115 (32.2%)
	D.	Rarely	N = 115 (32.2%)

Q6. How often do you eat dates?*	A.	Daily	N = 129 (36.1%)
	B.	Three or four times per week	N = 87 (24.4%)
	C.	Once or twice per week	N = 68 (19.0%)
	D.	Rarely	N = 73 (20.4%)

Q7. How often do you eat fruits except dates?*	A.	Daily	N = 16 (4.5%)
	B.	Three or four times per week	N = 68 (19.0%)
	C.	Once or twice per week	N = 144 (40.3%)
	D.	Rarely	N = 129 (36.1%)

Q8. How often do you eat fried food?	A.	Daily	N = 46 (12.9%)
	B.	Three or four times per week	N = 121 (33.9%)
	C.	Once or twice per week	N = 123 (34.5%)
	D.	Rarely	N = 67 (18.8%)

Q9. How often do you eat with family?	A.	Daily	N = 237 (66.4%)
	B.	Three or four times per week	N = 75 (21.0%)
	C.	Once or twice per week	N = 36 (10.1%)
	D.	Rarely	N = 9 (2.5%)

Q10. What type of food do you think you should eat to have a balanced nutrition?	A.	Mainly meat	N = 55 (15.4%)
	B.	Mainly vegetables	N = 66 (18.5%)
	C.	Meat, vegetables and other varieties of food	N = 213 (59.7%)
	D.	Others	N = 23 (6.4%)

Q11. Please state your smoking history?	A.	Current smoker	N = 32 (9.0%)
	B.	Ex-smoker	N = 15 (4.2%)
	C.	Never smoke	N = 310 (86.8%)

Q12. Did you ever drink alcohol?	A.	Yes	N = 15 (4.2%)
	B.	Never	N = 342 (95.8%)

* Dates were excluded from fruits in a separate question because they are a staple food in KSA.

### Correlations between anthropometry and eating habits

Correlating students anthropometric measurements with their eating habits (Tables 7, 8 and 9) revealed that both BMI and VFL had significant inverse correlations with the frequency of eating with family (P = 0.005 and 0.007 respectively). Similar correlations were also found between BMI and snacks consumption rate (P = 0.018), as well as, between VFL and the frequency of eating dates (P = 0.013).

**Table 7:** Correlations between BMI categories and eating habits [Total (Percent)].

Questions	Answer	BMI categories				Total (Percent)	P Value
				
Asked**	Levels**	Underweight	Normal	Overweight	Obese		
Q1.	A.	N = 2 (0.6%)	N = 83 (23.2%)	N = 27 (7.6%)	N = 19 (5.3%)	N = 131 (36.7%)	0.090
	B.	N = 16 (4.5%)	N = 122 (34.2%)	N = 51 (14.3%)	N = 37 (10.4%)	N = 226 (63.3%)	

Q2.	A.	N = 12 (3.4%)	N = 103 (28.9%)	N = 37 (10.4%)	N = 26 (7.3%)	N = 178 (49.9%)	0.075
	B.	N = 6 (1.7%)	N = 82 (23.0%)	N = 26 (7.3%)	N = 24 (6.7%)	N = 138 (38.7%)	
	C.	N = 0 (0.0%)	N = 15 (4.2%)	N = 8 (2.2%)	N = 6 (1.7%)	N = 29 (8.1%)	
	D.	N = 0 (0.0%)	N = 5 (1.4%)	N = 7 (2.0%)	N = 0 (0.0%)	N = 12 (3.4%)	

Q3.	A.	N = 0 (0.0%)	N = 25 (7.0%)	N = 12 (3.4%)	N = 3 (0.8%)	N = 40 (11.2%)	0.108
	B.	N = 13 (3.6%)	N = 106 (29.7%)	N = 46 (12.9%)	N = 34 (9.5%)	N = 199 (55.7%)	
	C.	N = 5 (1.4%)	N = 72 (20.2%)	N = 19 (5.3%)	N = 16 (4.5%)	N = 112 (31.4%)	
	D.	N = 0 (0.0%)	N = 2 (0.6%)	N = 1 (0.3%)	N = 3 (0.8%)	N = 6 (1.7%)	

Q4.	A.	N = 3 (0.8%)	N = 72 (20.2%)	N = 19 (5.3%)	N = 19 (5.3%)	N = 113 (31.7%)	0.018*
	B.	N = 9 (2.5%)	N = 55 (15.4%)	N = 19 (5.3%)	N = 6 (1.7%)	N = 89 (24.9%)	
	C.	N = 5 (1.4%)	N = 43 (12.0%)	N = 22 (6.2%)	N = 15 (4.2%)	N = 85 (23.8%)	
	D.	N = 1 (0.3%)	N = 35 (9.8%)	N = 18 (5.0%)	N = 16 (4.5%)	N = 70 (19.6%)	

Q5.	A.	N = 1 (0.3%)	N = 19 (5.3%)	N = 14 (3.9%)	N = 6 (1.7%)	N = 40 (11.2%)	0.753
	B.	N = 6 (1.7%)	N = 54 (15.1%)	N = 15 (4.2%)	N = 12 (3.4%)	N = 87 (24.4%)	
	C.	N = 6 (1.7%)	N = 65 (18.2%)	N = 23 (6.4%)	N = 21 (5.9%)	N = 115 (32.2%)	
	D.	N = 5 (1.4%)	N = 67 (18.8%)	N = 26 (7.3%)	N = 17 (4.8%)	N = 115 (32.2%)	

Q6.	A.	N = 8 (2.2%)	N = 83 (23.2%)	N = 24 (6.7%)	N = 14 (3.9%)	N = 129 (36.1%)	0.378
	B.	N = 5 (1.4%)	N = 48 (13.4%)	N = 22 (6.2%)	N = 12 (3.4%)	N = 87 (24.4%)	
	C.	N = 2 (0.6%)	N = 35 (9.8%)	N = 16 (4.5%)	N = 15 (4.2%)	N = 68 (19.0%)	
	D.	N = 3 (0.8%)	N = 39 (10.9%)	N = 16 (4.5%)	N = 15 (4.2%)	N = 73 (20.4%)	

Q7.	A.	N = 0 (0.0%)	N = 9 (2.5%)	N = 5 (1.4%)	N = 2 (0.6%)	N = 16 (4.5%)	0.479
	B.	N = 6 (1.7%)	N = 34 (9.5%)	N = 14 (3.9%)	N = 14 (3.9%)	N = 68 (19.0%)	
	C.	N = 6 (1.7%)	N = 91 (25.5%)	N = 31 (8.7%)	N = 16 (4.5%)	N = 144 (40.3%)	
	D.	N = 6 (1.7%)	N = 71 (19.9%)	N = 28 (7.8%)	N = 14 (3.9%)	N = 129 (36.1%)	

Q8.	A.	N = 2 (0.6%)	N = 27 (7.6%)	N = 10 (2.8%)	N = 7 (2.0%)	N = 46 (12.9%)	0.538
	B.	N = 9 (2.5%)	N = 61 (17.1%)	N = 33 (9.2%)	N = 18 (5.0%)	N = 121 (33.9%)	
	C.	N = 4 (1.1%)	N = 77 (21.6%)	N = 24 (6.7%)	N = 18 (5.0%)	N = 123 (34.5%)	
	D.	N = 3 (0.8%)	N = 40 (11.2%)	N = 11 (3.1%)	N = 13 (3.6%)	N = 67 (18.8%)	

Q9.	A.	N = 10 (2.8%)	N = 141 (39.5%)	N = 52 (14.6%)	N = 34 (9.5%)	N = 237 (66.4%)	0.005*
	B.	N = 3 (0.8%)	N = 49 (13.7%)	N = 15 (4.2%)	N = 8 (2.2%)	N = 75 (21.0%)	
	C.	N = 4 (1.1%)	N = 13 (3.6%)	N = 10 (2.8%)	N = 9 (2.5%)	N = 36 (10.1%)	
	D.	N = 1 (0.3%)	N = 2 (0.6%)	N = 1 (0.3%)	N = 5 (1.4%)	N = 9 (2.5%)	

Q10.	A.	N = 3 (0.8%)	N = 34 (9.5%)	N = 9 (2.5%)	N = 9 (2.5%)	N = 55 (15.4%)	0.986
	B.	N = 4 (1.1%)	N = 34 (9.5%)	N = 17 (4.8%)	N = 11 (3.1%)	N = 66 (18.5%)	
	C.	N = 10 (2.8%)	N = 123 (34.5%)	N = 47 (13.2%)	N = 33 (9.2%)	N = 213 (59.7%)	
	D.	N = 1 (0.3%)	N = 14 (3.9%)	N = 5 (1.4%)	N = 3 (0.8%)	N = 23 (6.4%)	

Q11.	A.	N = 1 (0.3%)	N = 19 (5.3%)	N = 6 (1.7%)	N = 6 (1.7%)	N = 32 (9.0%)	0.876
	B.	N = 0 (0.0%)	N = 9 (2.5%)	N = 3 (0.8%)	N = 3 (0.8%)	N = 15 (4.2%)	
	C.	N = 17 (4.8%)	N = 177 (49.6%)	N = 69 (19.3%)	N = 47 (13.2%)	N = 310 (86.8%)	

Q12.	A.	N = 0 (0.0%)	N = 8 (2.2%)	N = 3 (0.8%)	N = 4 (1.1%)	N = 15 (4.2%)	0.475
	B.	N = 18 (5.0%)	N = 197 (55.2%)	N = 75 (21.0%)	N = 52 (14.6%)	N = 342 (95.8%)	

* Indicate statistically significant correlation.

** Questions asked and answer levels were presented in Table 6.

**Table 8:** Correlations between BF% categories and eating habits [Total (Percent)].

Questions	Answer	BF% categories				Total (Percent)	P Value*
				
Asked**	Levels**	Low	Normal	High	Very High		
Q1.	A.	N = 2 (0.6%)	N = 57 (16.0%)	N = 23 (6.4%)	N = 49 (13.7%)	N = 131 (36.7%)	0.594
	B.	N = 9 (2.5%)	N = 92 (25.8%)	N = 37 (10.4%)	N = 88 (24.6%)	N = 226 (63.3%)	

Q2.	A.	N = 5 (1.4%)	N = 77 (21.6%)	N = 28 (7.8%)	N = 68 (19.0%)	N = 178 (49.9%)	0.464
	B.	N = 6 (1.7%)	N = 58 (16.2%)	N = 26 (7.3%)	N = 48 (13.4%)	N = 138 (38.7%)	
	C.	N = 0 (0.0%)	N = 10 (2.8%)	N = 6 (1.7%)	N = 13 (3.6%)	N = 29 (8.1%)	
	D.	N = 0 (0.0%)	N = 4 (1.1%)	N = 0 (0.0%)	N = 8 (2.2%)	N = 12 (3.4%)	

Q3.	A.	N = 0 (0.0%)	N = 17 (4.8%)	N = 9 (2.5%)	N = 14 (3.9%)	N = 40 (11.2%)	0.795
	B.	N = 7 (2.0%)	N = 83 (23.2%)	N = 30 (8.4%)	N = 79 (22.1%)	N = 199 (55.7%)	
	C.	N = 4 (1.1%)	N = 48 (13.4%)	N = 20 (5.6%)	N = 40 (11.2%)	N = 112 (31.4%)	
	D.	N = 0 (0.0%)	N = 1 (0.3%)	N = 1 (0.3%)	N = 4 (1.1%)	N = 6 (1.7%)	

Q4.	A.	N = 4 (1.1%)	N = 49 (13.7%)	N = 21 (5.9%)	N = 39 (10.9%)	N = 113 (31.7%)	0.275
	B.	N = 5 (1.4%)	N = 43 (12.0%)	N = 13 (3.6%)	N = 28 (7.8%)	N = 89 (24.9%)	
	C.	N = 2 (0.6%)	N = 33 (9.2%)	N = 12 (3.4%)	N = 38 (10.6%)	N = 85 (23.8%)	
	D.	N = 0 (0.0%)	N = 24 (6.7%)	N = 14 (3.9%)	N = 32 (9.0%)	N = 70 (19.6%)	

Q5.	A.	N = 1 (0.3%)	N = 10 (2.8%)	N = 7 (2.0%)	N = 22 (6.2%)	N = 40 (11.2%)	0.283
	B.	N = 5 (1.4%)	N = 41 (11.5%)	N = 15 (4.2%)	N = 26 (7.3%)	N = 87 (24.4%)	
	C.	N = 2 (0.6%)	N = 49 (13.7%)	N = 18 (5.0%)	N = 46 (12.9%)	N = 115 (32.2%)	
	D.	N = 3 (0.8%)	N = 49 (13.7%)	N = 20 (5.6%)	N = 43 (12.0%)	N = 115 (32.2%)	

Q6.	A.	N = 3 (0.8%)	N = 65 (18.2%)	N = 20 (5.6%)	N = 41 (11.5%)	N = 129 (36.1%)	0.483
	B.	N = 3 (0.8%)	N = 32 (9.0%)	N = 18 (5.0%)	N = 34 (9.5%)	N = 87 (24.4%)	
	C.	N = 2 (0.6%)	N = 23 (6.4%)	N = 12 (3.4%)	N = 31 (8.7%)	N = 68 (19.0%)	
	D.	N = 3 (0.8%)	N = 29 (8.1%)	N = 10 (2.8%)	N = 31 (8.7%)	N = 73 (20.4%)	

Q7.	A.	N = 0 (0.0%)	N = 5 (1.4%)	N = 3 (0.8%)	N = 8 (2.2%)	N = 16 (4.5%)	0.580
	B.	N = 3 (0.8%)	N = 27 (7.6%)	N = 8 (2.2%)	N = 30 (8.4%)	N = 68 (19.0%)	
	C.	N = 5 (1.4%)	N = 66 (18.5%)	N = 28 (7.8%)	N = 45 (12.5%)	N = 144 (40.3%)	
	D.	N = 3 (0.8%)	N = 51 (14.3%)	N = 21 (5.9%)	N = 54 (15.1%)	N = 129 (36.1%)	

Q8.	A.	N = 1 (0.3%)	N = 21 (5.9%)	N = 8 (2.2%)	N = 16 (4.5%)	N = 46 (12.9%)	0.935
	B.	N = 4 (1.1%)	N = 43 (12.0%)	N = 24 (6.7%)	N = 50 (14.0%)	N = 121 (33.9%)	
	C.	N = 4 (1.1%)	N = 54 (15.1%)	N = 18 (5.0%)	N = 47 (13.2%)	N = 123 (34.5%)	
	D.	N = 2 (0.6%)	N = 31 (8.7%)	N = 10 (2.8%)	N = 24 (6.7%)	N = 67 (18.8%)	

Q9.	A.	N = 6 (1.7%)	N = 97 (27.2%)	N = 43 (12.0%)	N = 91 (25.5%)	N = 237 (66.4%)	0.113
	B.	N = 2 (0.6%)	N = 40 (11.2%)	N = 11 (3.1%)	N = 22 (6.2%)	N = 75 (21.0%)	
	C.	N = 3 (0.8%)	N = 10 (2.8%)	N = 5 (1.4%)	N = 18 (5.0%)	N = 36 (10.1%)	
	D.	N = 0 (0.0%)	N = 2 (0.6%)	N = 1 (0.3%)	N = 6 (1.7%)	N = 9 (2.5%)	

Q10.	A.	N = 1 (0.3%)	N = 23 (6.4%)	N = 13 (3.6%)	N = 18 (5.0%)	N = 55 (15.4%)	0.819
	B.	N = 3 (0.8%)	N = 24 (6.7%)	N = 10 (2.8%)	N = 29 (8.1%)	N = 66 (18.5%)	
	C.	N = 6 (1.7%)	N = 91 (25.5%)	N = 35 (9.8%)	N = 81 (22.7%)	N = 213 (59.7%)	
	D.	N = 1 (0.3%)	N = 11 (3.1%)	N = 2 (0.6%)	N = 9 (2.5%)	N = 23 (6.4%)	

Q11.	A.	N = 0 (0.0%)	N = 13 (3.6%)	N = 6 (1.7%)	N = 13 (3.6%)	N = 32 (9.0%)	0.925
	B.	N = 0 (0.0%)	N = 6 (1.7%)	N = 3 (0.8%)	N = 6 (1.7%)	N = 15 (4.2%)	
	C.	N = 11 (3.1%)	N = 130 (36.4%)	N = 51 (14.3%)	N = 118 (33.1%)	N = 310 (86.8%)	

Q12.	A.	N = 0 (0.0%)	N = 5 (1.4%)	N = 3 (0.8%)	N = 7 (2.0%)	N = 15 (4.2%)	0.772
	B.	N = 11 (3.1%)	N = 144 (40.3%)	N = 57 (16.0%)	N = 130 (36.4%)	N = 342 (95.8%)	

* No statistically significant correlations were found.

** Questions asked and answer levels were presented in Table 6.

**Table 9:** Correlations between VFL categories and eating habits [Total (Percent)].

Questions	Answer	VFL categories		Total (Percent)	P Value
				
Asked**	Levels**	Normal	High		
Q1.	A.	N = 104 (29.1%)	N = 27 (7.6%)	N = 131 (36.7%)	0.666
	B.	N = 175 (49.0%)	N = 51 (14.3%)	N = 226 (63.3%)	

Q2.	A.	N = 142 (39.8%)	N = 36 (10.1%)	N = 178 (49.9%)	0.482
	B.	N = 105 (29.4%)	N = 33 (9.2%)	N = 138 (38.7%)	
	C.	N = 21 (5.9%)	N = 8 (2.2%)	N = 29 (8.1%)	
	D.	N = 11 (3.1%)	N = 1 (0.3%)	N = 12 (3.4%)	

Q3.	A.	N = 31 (8.7%)	N = 9 (2.5%)	N = 40 (11.2%)	0.060
	B.	N = 156 (43.7%)	N = 43 (12.0%)	N = 199 (55.7%)	
	C.	N = 90 (25.2%)	N = 22 (6.2%)	N = 112 (31.4%)	
	D.	N = 2 (0.6%)	N = 4 (1.1%)	N = 6 (1.7%)	

Q4.	A.	N = 87 (24.4%)	N = 26 (7.3%)	N = 113 (31.7%)	0.091
	B.	N = 77 (21.6%)	N = 12 (3.4%)	N = 89 (24.9%)	
	C.	N = 66 (18.5%)	N = 19 (5.3%)	N = 85 (23.8%)	
	D.	N = 49 (13.7%)	N = 21 (5.9%)	N = 70 (19.6%)	

Q5.	A.	N = 31 (8.7%)	N = 9 (2.5%)	N = 40 (11.2%)	0.694
	B.	N = 71 (19.9%)	N = 16 (4.5%)	N = 87 (24.4%)	
	C.	N = 86 (24.1%)	N = 29 (8.1%)	N = 115 (32.2%)	
	D.	N = 91 (25.5%)	N = 24 (9.7%)	N = 115 (32.2%)	

Q6.	A.	N = 110 (30.8%)	N = 19 (5.3%)	N = 129 (36.1%)	0.013*
	B.	N = 71 (19.9%)	N = 16 (4.5%)	N = 87 (24.4%)	
	C.	N = 48 (13.4%)	N = 20 (5.6%)	N = 68 (19.0%)	
	D.	N = 50 (14.0%)	N = 23 (6.4%)	N = 73 (20.4%)	

Q7.	A.	N = 12 (3.4%)	N = 4 (1.1%)	N = 16 (4.5%)	0.416
	B.	N = 51 (14.3%)	N = 17 (4.8%)	N = 68 (19.0%)	
	C.	N = 119 (33.3%)	N = 25 (7.0%)	N = 144 (40.3%)	
	D.	N = 97 (27.2%)	N = 32 (9.0%)	N = 129 (36.1%)	

Q8.	A.	N = 36 (10.1%)	N = 10 (2.8%)	N = 46 (12.9%)	0.882
	B.	N = 95 (26.6%)	N = 26 (7.3%)	N = 121 (33.9%)	
	C.	N = 98 (27.5%)	N = 25 (7.0%)	N = 123 (34.5%)	
	D.	N = 50 (14.0%)	N = 17 (4.8%)	N = 67 (18.8%)	

Q9.	A.	N = 186 (52.1%)	N = 51 (14.3%)	N = 237 (66.4%)	0.007*
	B.	N = 65 (18.2%)	N = 10 (2.8%)	N = 75 (21.0%)	
	C.	N = 24 (6.7%)	N = 12 (3.4%)	N = 36 (10.1%)	
	D.	N = 4 (1.1%)	N = 5 (1.4%)	N = 9 (2.5%)	

Q10.	A.	N = 42 (11.8%)	N = 13 (3.6%)	N = 55 (15.4%)	0.883
	B.	N = 50 (14.0%)	N = 16 (4.5%)	N = 66 (18.5%)	
	C.	N = 168 (47.1%)	N = 45 (12.6%)	N = 213 (59.7%)	
	D.	N = 19 (5.3%)	N = 4 (1.1%)	N = 23 (6.4%)	

Q11.	A.	N = 25 (7.0%)	N = 7 (2.0%)	N = 32 (9.0%)	0.899
	B.	N = 11 (3.1%)	N = 4 (1.1%)	N = 15 (4.2%)	
	C.	N = 243 (68.1%)	N = 67 (18.8%)	N = 310 (86.8%)	

Q12.	A.	N = 10 (2.8%)	N = 5 (1.4%)	N = 15 (4.2%)	0.271
	B.	N = 269 (75.4%)	N = 73 (20.4%)	N = 342 (95.8%)	

* Indicate statistically significant correlation.

** Questions asked and answer levels were presented in Table 6.

## Discussion

The purpose of this study was to assess overweight and obesity rates among male college students in KSA and to correlate their body weight status and composition with their eating habits. The current data demonstrated that more than one third of the students were above the normal body weight. Overweight students represented 21.8% of the sample whereas, 15.7% were obese. These findings were consistent with the results of similar studies in other Middle East and some Western countries. In Lebanon, the prevalence of overweight and obesity among male college students was 37.5% and 12.5%, respectively [20]. In Kuwait the corresponding percentages were 32% and 8.9% [29], while in the United States and the United Arab Emirates overweight and obese accounted for about 35% of the male college students [19,30,31]. In contrast, only 7.9% of Iranian male college students were above the normal body weight [32]. That rate decreased to 2.9% among Chinese college students with a percentage of obesity as low as 0.4 [23]. Despite the small sample sizes and the fact that self-reported height and weight were used in some of the above mentioned studies, their findings still reflect differences in the severity of obesity problems among young adults across nations.

Recently, obesity has been defined in terms of adiposity, rather than the relation of body weight to height and, in turn, body composition became a more desirable determinant of obesity than BMI [33,34]. That goes well with our results which confirmed that 38.4% of students are obese according to their BF% compared to 15.7% on basis of their BMI. The present work also demonstrated that the total body fat exceeded its normal values in more than half of the participants and the VFL was elevated in more than one fifth of them. Compared to those of similar studies, our results also revealed that normal, overweight and obese Saudi college students have on average more fat in their bodies than their Lebanese fellows [20], and their average BF% was higher than that in USA male college students of different ethnicities [35]. Moreover statistical analysis of the current data showed linear relationship between BF% and VFL among students. Health threatening values of VFL (≥ 10) were only found in subjects with very high BF% (≥ 25) and showed up in all obese and more than one fourth of overweight students. In literature, visceral fat has been closely linked to non-communicable diseases such as type II diabetes mellitus and coronary heart disease [36,37]. Therefore, urgent dietary management going hand in hand with regular medical follow up should be considered to overcome or, at least, minimize the risk of the above mentioned diseases in Saudi college students with high VFL [38].

The results of our study showed that most of the students have irregular meals with two main meals per day. With the exception of dates, which are a staple food in KSA, the majority of the students eat vegetables and fruits twice per week in maximum. As well, about half of the students eat fried foods three times per week in minimum. These habits need to be corrected using educational programs to promote healthy eating habits in KSA. On the other hand, most of the students take breakfast and snacks daily, eat with their families, are aware of the balanced nutrition and never smoke or drink alcohol. These habits ought to be encouraged. Comparing our results with equivalent studies from Lebanon and China [20,23], for students of the same gender, revealed diversity in eating habits among male college students in different societies. Most of Saudi students (63.3%) eat irregular meals while 64.6% of Lebanese and 81.6% of Chinese male students take regular meals. About half of Saudi students have breakfast daily compared to one third of Lebanese and two thirds of Chinese students. In KSA and Lebanon most of students (55.7% and 47.9% respectively) eat only two meals per day. In contrast, the vast majority of Chinese students (74.3%) eat meals thrice a day. Eating snacks was a daily habit in about one third of Saudi, half of Lebanese and only about one tenth of Chinese college students. Vegetables and fruits consumption was uncommon habit among Saudi students. On the other hand, 83.5% of Chinese and 56.3% of Lebanese male students consume vegetables three times or more per week. Moreover, 49% of Lebanese students eat fruits at the same rate. Most Saudi and Lebanese students eat with family, while most Chinese students eat alone.

It is well documented that vegetables and fruits are low in energy density because of their high water and fiber content. Therefore, adding them to a diet reduces its overall energy intake, thus, helping in weight management [39]. However, the current data showed insignificant (P > 0.05) correlation between BMI, BF% or VFL on one hand and vegetables and fruits consumption on the other hand. That could be explained by two factors; 1) inadequate intake of these foods and 2) the unhealthy habits entitled in their consumption. Eating raw vegetables and fruits in the course of a meal is uncommon among Saudi population. In addition, the vegetables content in most of the traditional Saudi dishes (e.g. Kabsa, Margog, Mandy) is too small to have an impact on the overall energy density of the diet. Moreover, fruits are usually taken as a dessert at the end of meals, thus, losing their "satiety effect" that tends to lower the overall energy intake of the diet. The term "snack" refers to all foods and drinks taken outside the context of the three main meals [40]. Although increased snacks consumption is often accused for increased prevalence of obesity, yet, a clear cut relation between snacking and BMI is still unsettled. Spanos and Hankey [41] examined the habitual meal and snaking patterns of university students and found no correlation between BMI and snacking. On the other hand, de Graaf [40] reported that snacks consumption may contribute to a positive energy balance and increased body weight. Contrarily, results of the present study revealed an inverse relationship between BMI and snacks eating rate. That can be explained by the high-calorie larger meals taken by the students in absence of snacks. This is supported by several epidemiological studies, as cited by Bellisle et al [42], which revealed an inverse relationship between habitual frequency of eating and BMI, leading to the assumption that increased eating of both meals and snacks frequency i.e. "nibbling meal pattern" helps in avoidance of obesity rather than the "gorging meal pattern". Moreover, a recent study on rats demonstrated that obesity development is associated with increased Calories per meal rather than per day, suggesting that the large size of meal, but not the overnutrition, could be responsible for obesity [43]. Similarly, significant inverse correlations were detected between both BMI and VFL, and the frequency of eating with family. This could be due to the fact that students, eating away from home, depend mainly on fast food high in Calories and fats and low in vegetables and fruits. This is supported by the results of earlier studies which reported that diets of the university students living away from the family are characterized by a number of undesirable practices affecting their healthy lifestyles. Significant decrease in the consumption of fruits, fresh and cooked vegetables, seafood and pulses together with increased intake of sugar and fast foods were the major dietary changes reported for university students living away from the family home. In addition, it has been suggested that the lack of experience in planning meals, and assuming responsibility for food purchasing and preparing for the first time are the main factors underlying the unhealthier dietary choices of these students [44,45]. Moreover, a unique finding in the present work was the significant inverse correlation between VFL and dates eating frequency. Dates are one of the main fruits frequently consumed by Saudis as snacks between meals and prior to main meals on social gatherings. It is well known that consuming the whole fruits promotes satiety and reduces energy intake at the next meal [39]. This may explain the inverse correlation between VFL and the frequency of dates consumption.

## Conclusions

In short, our findings showed high rates of overweight and obesity among male college students in KSA. Furthermore, BF% was elevated among more students, increasing the obesity prevalence by more than two times when used for defining adiposity rather than BMI. In contrast, the majority of the students possess normal VFL. High VFL's were encountered only in the extremely overweight and obese participants and, thus, can be used as a warning indicator for life threatening health problems associated with obesity such as diabetes and heart attack. Irregular and infrequent meals together with low vegetables and fruits intake were the most common unhealthy eating habits of the participants. Eating with family and frequent snacking were found to have a negative effect on BMI. Furthermore, VFL was inversely correlated with the frequency of both eating with family and dates' consumption. The university and college arenas represent the final opportunity for nutritional education of a large number of students. Our findings suggest the need for strategies and coordinated efforts at all levels (family, university, community and government) to reduce the tendency of overweight and obesity among college students and to promote healthy eating habits in our youth.

## Competing interests

The authors declare that they have no competing interests.

## Authors' contributions

ASA, AAF and NMA contributed equally to design the research protocol, conduct the data collection and analysis, and draft the paper. All authors read and approved the final manuscript.

## References

[B1] World Health Organization Obesity: preventing and managing the global epidemic. Report of a WHO consultation World Health Organ Tech Rep Ser 2000 894 1 253 http://whqlibdoc.who.int/trs/WHO_TRS_894.pdf (accessed September 1, 2010) 11234459

[B2] James WPT Jackson-Leach R Mhurchu CN Kalamara E Shayeghi M Rigby NJ Nishida C Rodgers A Ezzati M, Lopez AD, Rodgers A, Murray CJL Overweight and obesity (high body mass index) Comparative quantification of health risks: global and regional burden of disease attributable to selected major risk factors 2004 Geneva, World Health Organization 497 596

[B3] Yang P Zhou Y Chen B Wan HW Jia GQ Bai HL Wu XT Overweight, obesity and gastric cancer risk: Results from a meta-analysis of cohort studies Eur J Cancer 2009 45 16 2867 2873 10.1016/j.ejca.2009.04.019 19427197

[B4] Freedland SJ Wen J Wuerstle M Shah A Lai D Moalej B Atala C Aronson WJ Obesity Is a Significant Risk Factor for Prostate Cancer at the Time of Biopsy Urology 2008 72 1102 1105 10.1016/j.urology.2008.05.044 18722650 PMC3179687

[B5] Nguyen NT Magno CP Lane KT Hinojosa MW Lane JS Association of Hypertension, Diabetes, Dyslipidemia, and Metabolic Syndrome with Obesity: Findings from the National Health and Nutrition Examination Survey, 1999 to 2004 J Am Coll Surg 2008 207 928 934 10.1016/j.jamcollsurg.2008.08.022 19183541

[B6] Abbasi F Brown BW Lamendola C McLaughlin T Reaven GM Relationship Between Obesity, Insulin Resistance, and Coronary Heart Disease Risk J Am Coll Cardiol 2002 40 937 943 10.1016/S0735-1097(02)02051-X 12225719

[B7] Musaiger AO Al-Mannai MA Weight, height, body mass index and prevalence of obesity among the adult population in Bahrain Ann Hum Biol 2001 28 3 346 350 10.1080/030144601300119151 11393341

[B8] Al-Kandari YY Prevalence of obesity in Kuwait and its relation to sociocultural variables Obes Rev 2006 7 147 154 10.1111/j.1467-789X.2006.00231.x 16629871

[B9] Carter AO Saadi HF Reed RL Dunn EV Assessment of obesity, Lifestyle, and Reproductive Health Needs of Female Citizens of Al Ain, United Arab Emirates J Health Popul Nutr 2004 22 1 75 83 15190815

[B10] Al-Riyami AA Afifi MM Prevalence and correlates of obesity and central obesity among Omani adults Saudi Med J 2003 24 6 641 646 12847595

[B11] Alsaif MA Hakim IA Harris RB Alduwaihy M Al-Rubeaan K Al-Nuaim AR Al-Attas OS Prevalence and risk factors of obesity and overweight in adult Saudi population Nutr Res 2002 22 1243 1252 10.1016/S0271-5317(02)00439-6

[B12] Antonio G Chiara PA A Natural Diet Versus Modern Western Diets? A New Approach to Prevent "Well-Being Syndromes" Dig Dis Sci 2005 50 1 1 6 10.1007/s10620-005-1268-y 15712628

[B13] Amin TT Al-Sultan AI Ali A Overweight and obesity and their relation to dietary habits and socio-demographic characteristics among male primary school children in Al-Hassa, Kingdom of Saudi Arabia Eur J Nutr 2008 47 310 318 10.1007/s00394-008-0727-6 18677544

[B14] Mahfouz AA Abdelmoneim I Khan MY Daffalla AA Diab MM Al-Gelban KS Moussa H Obesity and Related Behaviors among Adolescent School Boys in Abha City, Southwestern Saudi Arabia J Trop Pediatr 2007 54 2 120 124 10.1093/tropej/fmm089 18039676

[B15] Al-Nuaim AA Bamgboye EA Al-Rubeaan KA Al-Mazrou Y Overweight and Obesity in Saudi Arabian Adult Population, Role of Sociodemographic Variables J Community Health 1997 22 3 211 223 10.1023/A:1025177108996 9178120

[B16] Al-Nozha MM Al-Mazrou YY Al-Maatouq MA Arafah MR Khalil MZ Khan NB Al-Marzouki K Abdullah MA Al-Khadra AH Al-Harthi SS Al-Shahid MS Al-Mobeireek A Nouh MS Obesity in Saudi Arabia Saudi Med J 2005 26 5 824 829 15951877

[B17] Al-Hazzaa HM Rising trends in BMI of Saudi adolescents: evidence from three national cross sectional studies Asia Pac J Clin Nutr 2007 16 3 462 466 17704028

[B18] El-Hazmi MA Warsy AS A Comparative Study of Prevalence of Overweight and Obesity in Children in Different Provinces of Saudi Arabia J Trop Pediatr 2002 48 3 172 177 10.1093/tropej/48.3.172 12164602

[B19] Huang TT Harris KJ Lee RE Nazir N Born W Kaur H Assessing Overweight, Obesity, Diet, and Physical Activity in College Students J Am Coll Health 2003 52 2 83 86 10.1080/07448480309595728 14765762

[B20] Yahia N Achkar A Abdallah A Rizk S Eating habits and obesity among Lebanese university students Nutr J 2008 7 32 10.1186/1475-2891-7-32 18973661 PMC2584644

[B21] Rasheed P Abou-Hozaifa BM Kahn A Obesity among young Saudi female adults: a prevalence study on medical and nursing students Public Health 1994 108 4 289 294 10.1016/S0033-3506(94)80008-1 8066174

[B22] Sakamaki R Amamoto R Mochida Y Shinfuku N Toyama KA A comparative study of food habits and body shape perception of university students in Japan and Korea Nutr J 2005 4 31 10.1186/1475-2891-4-31 16255785 PMC1298329

[B23] Sakamaki R Toyama K Amamoto R Liu CJ Shinfuku N Nutritional knowledge, food habits and health attitude of Chinese university students -a cross sectional study-Nutr J 2005 4 4 10.1186/1475-2891-4-4 15703071 PMC553986

[B24] Bosy-Westphal A Later W Hitze B Sato T Kossel E Glüer CC Heller M Müller MJ Accuracy of Bioelectrical Impedance Consumer Devices for Measurement of Body Composition in Comparison to Whole Body Magnetic Resonance Imaging and Dual X-Ray Absorptiometry Obes Facts 2008 1 319 324 20054195 10.1159/000176061 PMC6452160

[B25] Gibson RS Nutritional assessment: a laboratory manual 1993 Oxford university press; New York, USA

[B26] National Institutes of Health/National Heart and Blood Institute Clinical Guidelines on the identification, evaluation, and treatment of overweight and obesity in adults The Evidence Report 1998 4083 1 228 http://www.nhlbi.nih.gov/guidelines/obesity/ob_gdlns.pdf (accessed September 1, 2010) 9813653

[B27] Gallagher D Heymsfield SB Heo M Jebb SA Murgatroyd PR Sakamoto Y Healthy percentage body fat ranges: an approach for developing guidelines based on body mass index Am J Clin Nutr 2000 72 694 701 10966886 10.1093/ajcn/72.3.694

[B28] Omron Healthcare Co Ltd Body Composition Monitor BF500 Instruction Manual http://www.pro2move.nl/images/HBF500%20gebruiksaanwijzing.pdf (accessed September 1, 2010)

[B29] Al-Isa AN Obesity among Kuwait University students: an explorative study J R Soc Promot Health 1999 119 4 223 227 10.1177/146642409911900404 10673842

[B30] Lowry R Galuska DA Fulton JE Wechsler H Kann L Collins JL Physical activity, food choice, and weight management goals and practices among US college students Am J Prev Med 2000 18 18 27 10.1016/S0749-3797(99)00107-5 10808979

[B31] Musaiger AO Lloyd OL Al-Neyadi SM Bener AB Lifestyle factors associated with obesity among male university students in the United Arab Emirates Nutr Food Sci 2003 33 4 145 147 10.1108/00346650310488480

[B32] Nojomi M Najamabadi S Obesity among university students, Tehran, Iran Asia Pac J Clin Nutr 2006 15 4 516 20 17077068

[B33] Seidell JC Flegal KM Assessing obesity: classification and epidemiology Br Med Bull 1997 53 2 238 252 9246834 10.1093/oxfordjournals.bmb.a011611

[B34] Frankenfield DC Rowe WA Cooney RN Smith JS Becker D Limits of Body Mass Index to Detect Obesity and Predict Body Composition Nutrition 2001 17 26 30 10.1016/S0899-9007(00)00471-8 11165884

[B35] Koutoubi S Huffman FG Body Composition Assessment and Coronary Heart Disease Risk Factors among College Students of Three Ethnic Groups J Nati Med Assoc 2005 97 6 784 791 PMC2569494 16035576

[B36] Gabriely I Ma XH Yang XM Atzmon G Rajala MW Berg AH Scherer P Rossetti L Barzilai N Removal of visceral fat prevents insulin resistance and glucose intolerance of aging Diabetes 2002 51 10 2951 2958 10.2337/diabetes.51.10.2951 12351432

[B37] Marques MD Santos RD Parga JR Rocha-Filho JA Quaglia LA Miname MH Ávila LF Relation between visceral fat and coronary artery disease evaluated by multidetector computed tomography Atherosclerosis 2010 209 2 481 486 10.1016/j.atherosclerosis.2009.10.023 19922936

[B38] Shoji K Maeda K Nakamura T Funahashi T Matsuzawa Y Shimomura I Measurement of visceral fat by abdominal bioelectrical impedance analysis is beneficial in medical checkup Obes Res Clin Pract 2008 2 269 275 10.1016/j.orcp.2008.09.001 24351854

[B39] Rolls BJ Ello-Martin JA Tohill BC What Can Intervention Studies Tell Us about the Relationship between Fruit and Vegetables Consumption and Weight Management? Nutr Rev 2004 62 1 1 17 10.1111/j.1753-4887.2004.tb00001.x 14995052

[B40] de Graaf C Effects of snacks on energy intake: An evolutionary perspective Appetite 2006 47 18 23 10.1016/j.appet.2006.02.007 16675059

[B41] Spanos D Hankey CR The habitual meal and snacking patterns of university students in two countries and their use of vending machines J Hum Nutr Diet 2010 23 102 107 10.1111/j.1365-277X.2009.01005.x 19943844

[B42] Bellisle F McDevitt R Prentice AM Meal frequency and energy balance Br J Nutr 1997 77 Suppl 1 S57 S70 10.1079/BJN19970104 9155494

[B43] Furnes MW Zhao CM Chen D Development of Obesity is Associated with Increased Calories per Meal Rather than per Day. A Study of High-Fat Diet-Induced Obesity in Young Rats Obes Surg 2009 19 1430 1438 10.1007/s11695-009-9863-1 19506986

[B44] Papadaki A Hondros G Scott JA Kapsokefalou M Eating habits of University students living at, or away from home in Greece Appetite 2007 49 169 176 10.1016/j.appet.2007.01.008 17368642

[B45] Kremmyda LS Papadaki A Hondros G Kapsokefalou M Scott JA Differentiating between the effect of rapid dietary acculturation and the effect of living away from home for the first time, on the diets of Greek students studying in Glasgow Appetite 2008 50 455 463 10.1016/j.appet.2007.09.014 17997195

